# Hemostatic agents in endodontic surgery of maxillary molars: A randomized controlled pilot study of polytetrafluoroethylene (PTFE) strips as an adjunct to epinephrine impregnated gauze versus aluminum chloride

**DOI:** 10.4317/medoral.23652

**Published:** 2020-07-19

**Authors:** David Peñarrocha-Oltra, David Soto-Peñaloza, Miguel Peñarrocha-Diago, Juan Cervera-Ballester, Guillermo Cabanes-Gumbau, María Peñarrocha-Diago

**Affiliations:** 1 DDS, PhD. Associate Professor, Department of Stomatology, Valencia University Medical and Dental School, Valencia, Spain; 2 DDS, MS, PhD. Master in Oral Surgery and Implantology. Valencia University Medical and Dental School, Valencia, Spain; 3 MD, PhD. Chairman of Oral Surgery and Director of the Master in Oral Surgery and Implantology, Valencia University Medical and Dental School, Valencia, Spain; 4 DDS, MS. Collaborative Professor of the Master in Oral Surgery and Implantology. Valencia University Medical and Dental School, Valencia, Spain; 5 MD, DDS, MS, PhD. Full Professor of Oral Surgery, Department of Stomatology, Valencia University Medical and Dental School, Valencia, Spain

## Abstract

**Background:**

Hemostasis is of critical importance in endodontic surgery. Studies on bleeding control in maxillary molars are scarce. The present study compares the efficacy of two hemostatic techniques in controlling bleeding in endodontic surgery.

**Material and Methods:**

A randomized two-arm pilot study involving 30 patients with peri-radicular lesions in maxillary molars (first and second molars) was carried out including the following hemostatic agents: polytetrafluoroethylene (PTFE) strips as an adjunct to epinephrine impregnated gauze (test group; n = 15) and aluminum chloride (Expasyl™) (control; n = 15). Bleeding control was independently assessed by the surgeon and by two blinded observers before and after application of the hemostatic agent, and was classified as either adequate (complete bleeding control) or inadequate (incomplete bleeding control).

**Results:**

Bleeding control was similar in both groups. Simple binary logistic regression analysis failed to identify variables affecting bleeding control. Only the height of the keratinized mucosal band (≥ 2 mm) suggested a decreased risk of inadequate bleeding control of up to 89% (OR=0.11; *p*=0.06).

**Conclusions:**

No difference in the efficacy of bleeding control was observed between PTFE strips as an adjunct to epinephrine impregnated gauze and aluminum chloride in maxillary molars.

** Key words:**PTFE-strips, aluminum chloride, endodontic surgery, epinephrine, hemostasis, hemostatic agent, molars.

## Introduction

Endodontic surgery could be considered an advisable and healthy option for saving a tooth. Such surgery is reported to yield healing rates of up to 82% at 10 years, with an 86% probability of remaining healed after 10 years in those teeth found to be healed at one year postsurgery (1). Hemostasis control is an essential element in endodontic surgery, ensuring a proper environment for retrograde filling; it affords better visibility of the surgical field and improved ergonomics, and contributes to lessen postsurgical bleeding and swelling (2).

In maxillary molars (first and second molars), endodontic surgery is a challenging procedure due to difficult access, a limited field of view, and the intricate anatomical structures in the vicinity of these antral teeth that may affects the outcomes of endodontic surgery and complications occurrence (e.g., proximity to the sinus floor, the presence of isthmuses, odontogenic sinusitis, and presence vascular bone channel)(3-6). The latter of especial interest during the preparation of the access window, because of existence of vascular bone channel closer to the mesiobuccal root apex of maxillary molars, when are buccally positioned (6).

At present, endodontic surgery in this scenario is often avoided because of the difficulties involved, particularly in the case of teeth with affected palatal roots (7). Such reluctance is related to a lack of experience but also to technical difficulties in securing satisfactory hemostasis.

In order to achieve treatment success (periapical tissue healing), strict surgical steps must be followed: surgical exposure of the root-end, debridement of pathological tissue, root-end resection, retrograde cavity preparation, retrograde filling and verification of root integrity (8). Notwithstanding, the better the bleeding control, the better the visibility of the surgical field for satisfactory filling, favoring cement setting, and for corroborating possible root fractures.

Several hemostatic agents / techniques has been introduced in endodontic surgery (e.g., bone wax, collagen membranes, aluminum chloride, ferric sulfate, epinephrine). It is known that an ideal hemostatic agent for endodontic microsurgery must have a quick hemostatic effect, must be easy to handle, should be biocompatible, and must not undermine natural bony crypt healing and the surrounding tissues (9).

Evidence suggests that some hemostatic agents may impair wound healing, exerting cytotoxic effects upon bone cells and tissue remodeling, reflected by chronic inflammation, foreign body reactions and inflammatory tissue responses (10-13). In particular, this is the case of paste-based hemostatic agents (e.g., bone wax, aluminum chloride), which are difficult to thoroughly remove, especially in maxillary molars. On the other hand, such problems are attenuated if the superficial bone layer is removed using rotary instruments, with abundant saline irrigation (14).

To date, few clinical trials have assessed the efficacy of hemostatic agents (15-17). Although there is no consensus regarding the ideal hemostatic option, a well-powered clinical trial showed aluminum chloride paste (Expasyl™, Produits Dentaires Pierre Rolland, Merignac, France) to afford significantly better bleeding control versus epinephrine alone (*p*<0.05) (18).

A recent pilot study on polytetrafluoroethylene (PTFE) strips as an adjunct to epinephrine-impregnated gauzes for securing hemostasis has reported results similar to those obtained with aluminum chloride paste, but referred to anterior teeth (second premolar to the second premolar of the upper maxilla) (19). Polytetrafluoroethylene strips exert a mechanical hemostatic barrier effect in the same way as other materials (e.g., bone wax), and can be autoclaved without losing their physical properties or barrier function (20). Polytetrafluoroethylene is a biocompatible material that is easy to handle and is used in restorative dentistry and guided bone regeneration as membrane material (20,21). It is used as an adjunct to epinephrine-impregnated gauzes because it is a cheap and affordable alternative that does not leave hemostatic material traces that may impair wound healing, affording a synergistic effect upon bleeding control (19).

The proximity of maxillary molars to the sinus membrane has been associated with significantly increased odds of sinus pathology (thickening of sinus membrane and odontogenic sinusitis) (3). Moreover, this close relationship may increase the risk of bleeding during endodontic surgery of maxillary molars, requiring efficient bleeding control. The possibility of having residues of paste-based hemostatic agents (like Expasyl™) in proximity to the sinus membrane or even intruding in the maxillary sinus should be considered, as it could trigger sinus membrane reactions. In this sense, the seek for materials that optimize hemostasis in endodontic surgery but are easily removed or physiologically resorbed is an issue of utmost importance. Thus, the use of PTFE strips adjunct to epinephrine impregnated gauzes makes particular sense as an alternative in maxillary molars.

To date, no clinical studies have evaluated the use of PTFE strips as an adjunct to hemostasis in endodontic surgery of maxillary molars. The present clinical study was designed to test the null hypothesis that there are no differences in bleeding control with PTFE strips as an adjunct to epinephrine-impregnated gauze versus aluminum chloride paste (ExpasylTM) in endodontic surgery of maxillary molars.

## Material and Methods

- Study design

A randomized efficacy pilot study was conducted according to the CONSORT (Consolidated Standards of Reporting Trials) statement (http://www.consort-statement.org) (22) and in agreement with the Declaration of Helsinki [1975] regarding biomedical research involving human subjects, as revised in Fortaleza [2013] (23). The study protocol was approved by the local Ethics Committee of the University of Valencia (Protocol ref.: H1481198441228).

- Patient selection 

Patients requiring endodontic surgery of maxillary first and second molars were recruited over 24 months from May 2017 to June 2019 in the Oral Surgery and Implantology Unit (Department of Stomatology, Valencia University Medical and Dental School, Valencia, Spain).

The following inclusion criteria were applied:

1. Good general condition (ASA score 1 or 2).

2. Single teeth needing endodontic surgery, with a lesion of strictly endodontic origin (chronic apical periodontitis).

3. Bone defects between 8-12 mm in diameter as determined from periapical radiographs.

4. Unfeasible or previously failed nonsurgical re-treatment.

5. An apical root canal devoid of a post over a length of at least 6 mm.

6. No spontaneous pain or swelling at the time of the operation.

The following exclusion criteria were applied: vertical root fracture, through-and-through lesions, perforation of lateral canal walls, previous traumatic injuries, contiguous tooth with severe periodontal disease, periodontal bone loss (probing depth > 5 mm), inadequate occlusion or parafunctions, and patients with non-controlled mental disorders. Only posterior teeth (first and second molars) were considered. Patients were considered for inclusion after inspection by a clinician with expertise in endodontic surgery (JCB).

- Sample size estimation

For an alpha value of 0.05 and a beta value of 0.2 (80%) in order to detect an odds ratio (OR) = 3.75 through binary logistic regression analysis (representing a huge effect size difference of 25% at rates of effectiveness between hemostatic techniques), 34 teeth were considered (17 per group). Because of dropouts, the present pilot study includes 30 patients/teeth (15 per group). The post-hoc power analysis is estimated at 74.2% (beta=0.25) under the same previous conditions.

- Sequence generation and allocation concealment

Allocation to either the test group (PTFE strips + epinephrine) or the control group (aluminum chloride) was made based on a computer-generated randomization list. To ensure similar patient distribution between treatment groups, computer-generated permuted block sequencing was performed, owing to the small number of individuals in this pilot study. Allocation concealment was implemented using opaque envelopes containing the assigned intervention that was picked up from a container and notified to the surgeons (MiPD or DPO) by an independent researcher (MaPD) after mucoperiosteal flap release.

- Surgical procedure

Prophylactic antibiotic treatment with 2 g of amoxicillin (or 600 mg of clindamycin in the case of allergy to amoxicillin) was prescribed to all participants one hour before surgery. The surgeries were carried out by two oral surgeons (MiPD and DPO). Infiltrative local anesthesia with 4% articaine and 1:100,000 epinephrine (Inibsa; Lliça de Vall, Barcelona, Spain) was provided. Depending on the teeth features, the flap designs were raised with a paramarginal or sulcular incision using a 15c surgical blade. The entire surgical procedure was conducted using an endodontic microscope (Extaro 300, Carl-Zeiss Meditec AG, Jena, Germany). After mucoperiosteal flap release, an osteotomy was carried out with abundant sterile saline solution using a 0.27-mm diameter round tungsten-carbide burr (Jota AG, Rüthi SG, Switzerland) mounted in a 1:1 handpiece (W&H Dentalwerk, Burmoss, Austria). Then, granulomatous inflammatory tissue surrounding the root apex was debrided using manual curettes. In the test group, hemostasis was achieved using sterile epinephrine-impregnated gauze and autoclaved PTFE strips. The PTFE strips were set within the bony crypt using forceps, and then compacted using an amalgam ball burnisher - plugger. In the control group, a paste-based hemostatic agent (aluminum chloride, Expasyl™) was locally applied within the bony crypt for two minutes and then removed with the help of a dental curette and abundant sterile saline solution. The integrity of the root-ends was inspected employing a rigid endoscope (Karl Storz-Endoskope, Tuttlingen, Germany). Three-millimeter resection of the apical portion was performed using ultrasonic instruments. Then, the root-ends was instrumented to produce a retrograde cavity 3 mm in depth using ultrasonic retro-tips (Piezomed, W&H Dentalwerk Bürmoos GmbH, Bürmoos, Salzburg, Austria), which was retro-filled with mineral trioxide aggregate cement (MTA; Dentsply Tulsa Dental Specialties, Tulsa, OK, USA). Finally, the rigid endoscope was again used to inspect the quality of the retrograde filling. Tension-free flap closure was performed in both groups using interrupted stitches with the same 6/0 suture material (Polinyl, Sweden & Martina, Carrare, Italy).

- Postoperative instructions

All patients received ice packs in the immediate postoperative period for 20 minutes. Participants were advised to avoid smoking, tooth brushing or cleansing around the surgical area during 24 hours. Rinses with 0.12% chlorhexidine twice a day for 7 days and 400 mg of ibuprofen every 8 hours for three days, were prescribed. Sutures were removed one week postsurgery.

- Data collection

**Figure 1:**
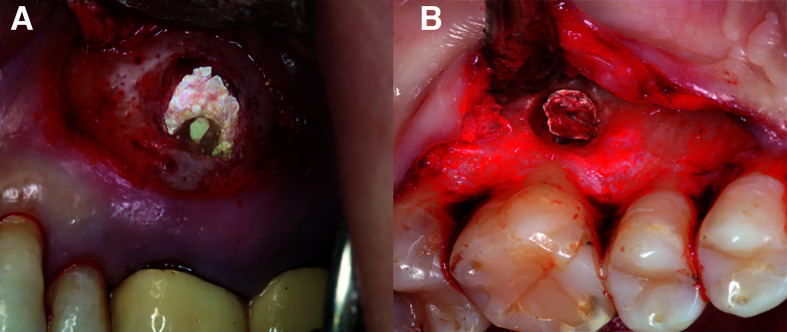
Hemostasis: A) adequate, B) inadequate.

Primary outcome: Assessment of intraoperative bleeding control after hemostasis with two distinct 

techniques. Secondary outcomes: Assessment of the effect of covariables and potential effect modifiers (e.g., hemostatic agent, patient gender, age, preoperative pain, presence of fistula, smoking habit, tooth type, root type and gingival biotype) upon bleeding control. The surgeon (either MiPD or DPO) and two blinded evaluators (DSP and GC), neither aware of nor involved in patient selection or the treatment provided, assessed the hemostasis achieved independently, and classified it as either adequate or inadequate (Fig. 1), in accordance to previous reports (16,24):

1. Adequate hemostasis: complete bleeding control, with a dry surgical field after application of the hemostatic material (Fig. 1).

2. Inadequate hemostasis: slight but apparent intermittent bleeding persisting after application of the hemostatic material (allowing the root-end filling procedure to be completed) (Fig. 1).

Bleeding control was evaluated intraoperatively by the surgeon and postoperatively by the two blinded evaluators using photographs. The bony crypts were photographed (Canon EOS 70D, Canon Macro Ring Lite MR-14EX, Canon EF 100 mm f/2.8 Macro USM; Tokyo, Japan) before application and after removal of the hemostatic agents for the records of the blinded outcome assessors (DSP and GC). A PDF file containing the clinical photographs (Adobe Acrobat Reader DC; Adobe Systems Inc, San Jose, CA, USA) was submitted to the two blinded evaluators for viewing on a 21.5-inch monitor (iMac; Apple, Cupertino, CA, USA) with a screen resolution of 4096 x 2304 pixels. All the observers (MiPD, DPO, DSP and GC) were blinded to the evaluation of the rest. The sequentially-numbered datasheets with the bleeding control records of each evaluator were collected, transferred and labeled using a blinded code corresponding to each advisor in an Excel spreadsheet by an independent researcher (JCB). This file was shared electronically for analysis by a blinded statistician.

- Descriptive and inferential analyses

Analyses were performed by a blinded statistician not aware of the allocation groups. Calculation was made of the mean, standard deviation, median and range for quantitative variables, and of the absolute and relatives frequencies for categorical variables. The Student t-test and chi-squared test were used for the comparison of sample characteristics between the groups. The tooth was considered as the unit of analysis. The level of statistical significance was set at 5% (α=0.05). Simple binary logistic regression analysis was performed for each parameter independently, with calculation of the odds ratio (OR) and 95% confidence interval (95%CI), to explore associations between independent variables according to the hemostatic group involved. In the case of continuous data, e.g., patient age, we adopted a dummy dichotomous variable approach, with the definition of cut-off values (≤ 50 and > 50 years). The intraclass correlation coefficient (ICC) and corresponding 95%CI were estimated based on mean-rating (k=4) evaluators and the consistency among them, through a two-way mixed-effects model in accordance with the guidelines of Shrout and Fleiss [1979] (25,26). The correlation coefficient was interpreted using the Landis and Koch scale (27). The SPSS version 22.0 statistical package (SPSS Inc., Chicago, IL, USA) was used throughout.

## Results

- Patient selection and baseline data

**Figure 2:**
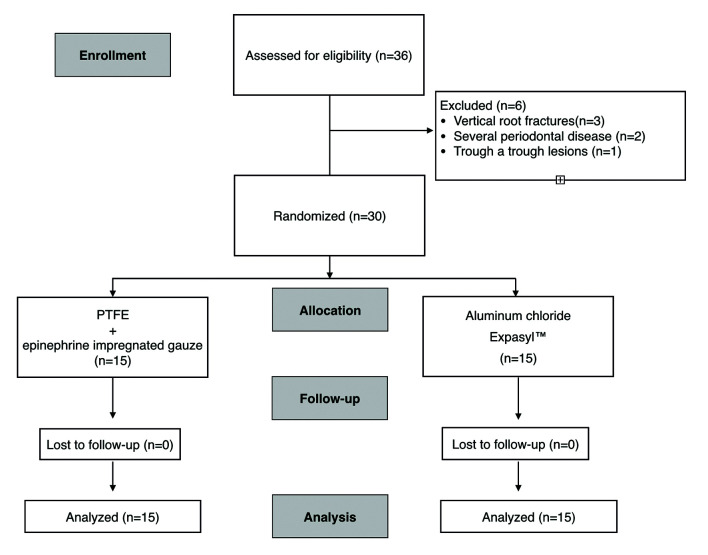
CONSORT flowchart of patients selection.

Of the 36 patients initially recruited, 6 were excluded: three had vertical root fractures, two presented severe periodontal disease of a contiguous tooth, and one presented a through-and-through lesion. A total of 30 individuals comprising 30 teeth were thus randomly allocated to either the test group (PTFE strips + epinephrine) or the control group (Expasyl™)(15 individuals/teeth in each group) (Fig. 2). The patient characteristics were comparable between groups, with no significant differences (Table 1). The mean age of the study sample was 48.010.8 years (8 males and 22 females). Eight patients reported preoperative pain, four presented fistulas, 9 were smokers, 5 had an affected palatal root, and 13 had a thin keratinized mucosa height (Table 1). Persistent apical lesion after root canal retreatment was the most common indication of endodontic surgery.

**Table 1:**
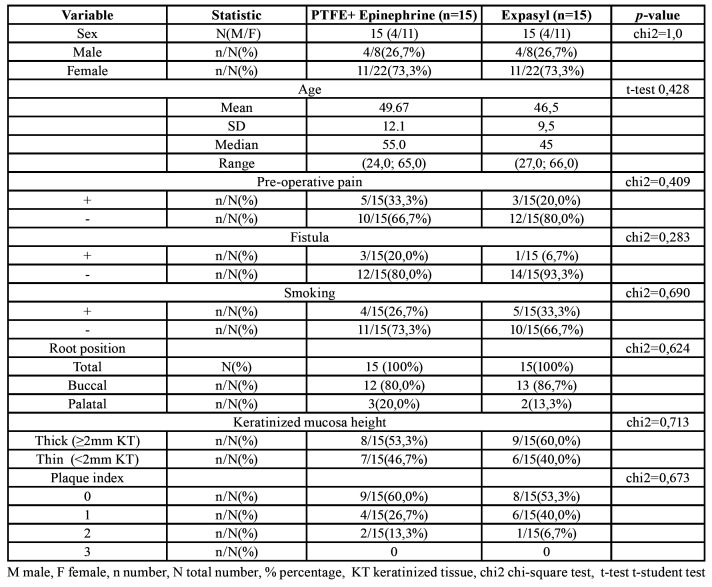
Study sample characteristics.

- Hemostatic effect

**Figure 3:**
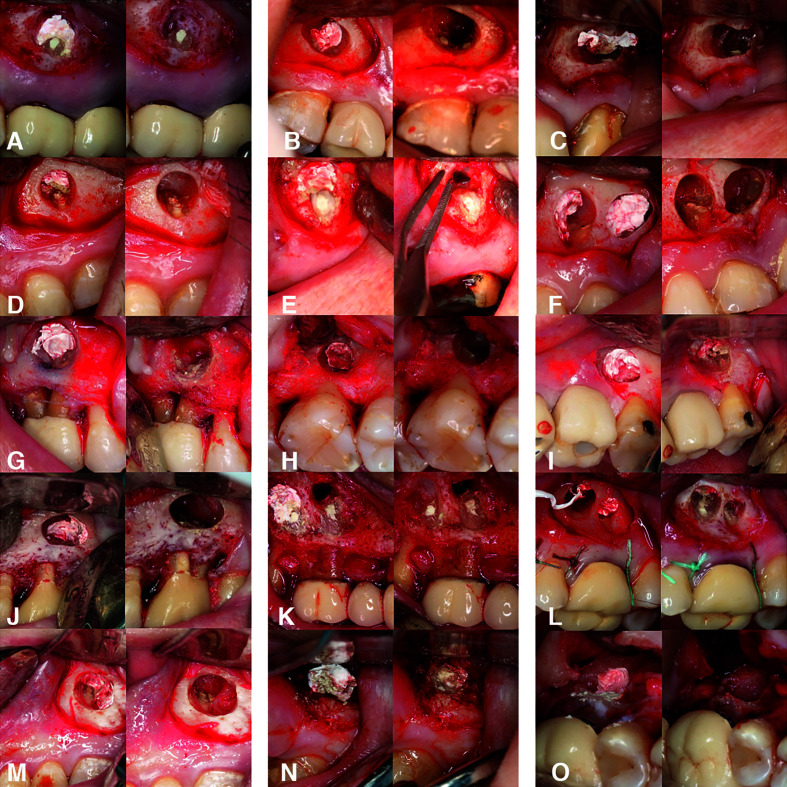
Endodontic surgery hemostasis in the PTFE strips + epinephrine group. Two images are shown of each of the 15 cases from A to O (left and right sides). Left side, initial bleeding; right side, bleeding control reached after hemostatic agent removal.

**Figure 4:**
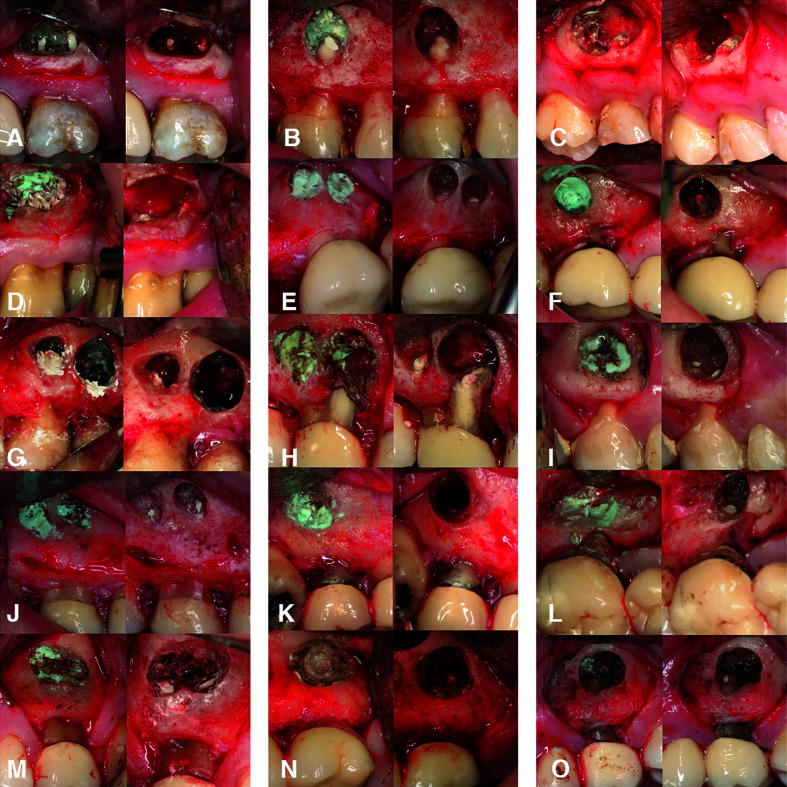
Endodontic surgery hemostasis in the Expasyl™ group. Two images are shown of each of the 15 cases from A to O (left and right sides). Left side, initial bleeding; right side, bleeding control reached.

In the PTFE strips + epinephrine group, adequate hemostasis was achieved in 11 of the 15 cases (73.3%) (Fig. 3), while in the control group (Expasyl™) adequate hemostasis was achieved in 10 of the 15 cases (66.7%) (Fig. 4). There were no statistically significant differences between the hemostatic agents in terms of bleeding control (*p*=0.690) (Table 2). Inter-rater consistency according to the ICC was 0.972 with 95%CI (0.95 to 0.99) (*p*=0.001). The level of agreement was considered almost perfect according to the scale proposed by Landis and Koch (27). The included patient variables and risk factors had no significant impact upon bleeding control, though the height of keratinized mucosa showed a certain trend towards statistical significance (*p*=0.064), suggesting a possible decrease in the risk (beta = -2,211) of inadequate bleeding of up to 89% (OR=0.11) (Table 3).

**Table 2:**
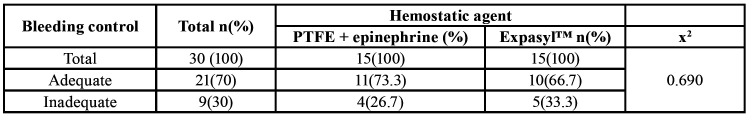
Efficacy of bleeding control according to the hemostatic agent used.

**Table 3:**
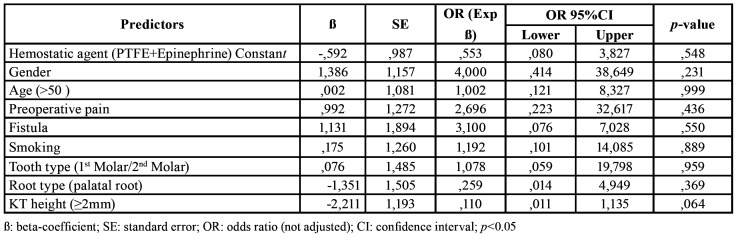
Simple binary logistic regression analysis adjusted to potential effect moderators (covariables).

## Discussion

The present pilot study was designed to test the null hypothesis that there are no differences in bleeding control in endodontic surgery between PTFE strips as an adjunct to epinephrine-impregnated gauze and aluminum chloride paste (ExpasylTM), in maxillary molars. No significant differences were observed between the hemostatic techniques in the maxillary molars, suggesting that PTFE strips as an adjunct to epinephrine-impregnated gauze afford bleeding control similar to that achieved with aluminum chloride paste in the present study sample.

The search for an ideal hemostatic agent / technique is of utmost importance, for as is well known, common hemostatic agents locally applied within the bony crypts (e.g., bone wax, Expasyl™ plus ferric sulfate or electrocauterization) may impair wound healing and induce foreign body reactions or cytotoxic effects upon bone cells, producing necrosis and inflammatory cell infiltration (10–13) that subsequently impair the innate bone repair potential (14). Also, hemostatic material traces remaining within the bony crypt exacerbate the postoperative inflammatory response. This is a relevant fact, since paste-based hemostatic agents (e.g., aluminum chloride, bone wax) can prove difficult to remove entirely, because of adherence to the bony crypt walls, the position of the tooth and defect, or retention by the intricate root anatomy (e.g., first and second molars of the upper maxilla). Moreover, in maxillary molars, the use of paste-based hemostatic agents should be avoided because of the risk of intrusion in the maxillary sinus and the chance of its incomplete removal.

A relevant fact, since this close anatomical relation has been proven to increase the odds of sinus pathology of dental origin (3).

An option for overcoming the deleterious effects of the material traces adhered within the bony crypt is to refresh the bone with rotary instruments and copious irrigation, thereby ensuring complete removal. This strategy is not always useful, however.

Ensuring natural healing after endodontic surgery is of paramount importance for treatment success over the long term. In this regard, an endodontically treated tooth has an 86% probability of remaining healed after 10 years if the lesion is found to be healed at one year post-surgery (1). The ideal strategy is therefore not to leave hemostatic material traces, and to simultaneously provide an adequate hemostatic effect. The need for such a strategy arises because previous trials have found that epinephrine alone (7), or electrocauterization (28), are not superior to aluminum chloride paste in relation to bleeding control. The search for a hemostatic agent/technique that does not leave material traces or affect wound healing therefore continues. Seeking an alternative, we previously reported the use of autoclavable PTFE strips as an adjunct to epinephrine-impregnated gauze, based on the rationale that it leaves no material traces and may exert a synergistic effect upon the hemostatic properties of epinephrine-impregnated gauze alone (19).

This proposal was formally tested in a previous randomized pilot study centered on the anterior zone of the upper maxilla (second premolar to second premolar). The mentioned study found no differences in bleeding control with respect to aluminum chloride paste (19). The present study attempted to establish the same comparison but in the posterior maxilla. Although the difficult access and intricate anatomical features characterizing the posterior sector pose a challenge, PTFE strips as an adjunct to epinephrine-impregnated gauze represents a feasible option that does not leave material traces capable of impairing wound healing, providing adequate bleeding control comparable to that afforded by aluminum chloride paste.

However, some limitations of our study need to be mentioned. The main problem is its limited sample size, since this is a pilot study. Our main aim was to assess the hemostatic effect; hence, there is no information regarding other variables, such as long-term success rates, radiological healing, or patient-reported outcomes, which are relevant when deciding which hemostatic agent to use. Further studies are warranted, involving larger sample sizes and long-term follow-up, with the assessment of radiological healing and patient-reported outcome measures.

Despite the limitations, the data obtained in the present pilot study could be useful for enhancing bleeding control in endodontic surgery in maxillary molars. The described approach using PTFE strips as an adjunct to epinephrine-impregnated gauze allows clinicians to individualize treatment, and is inexpensive and easy to implement. It also offers good handling performance at sites with intricate anatomical features, such as multi-rooted tooth, or in the vicinity of bleeding anatomical structures such as the maxillary sinus, and to avoid exacerbating the inflammatory response of sinus mucosa due to material incomplete removal or intrusion. The PTFE strips act as a mechanical barrier that can be easily removed when bleeding control is no longer necessary, without leaving material traces. Another strong point of the study is the use by expert surgeons of microscopic and endoscopic techniques that allow the assessment of root integrity, bleeding control and the quality of retrograde filling.

## Conclusions

No differences in the efficacy of bleeding control were observed between PTFE strips as an adjunct to epinephrine-impregnated gauze and aluminum chloride (Expasyl™) in maxillary molars.
